# Assessing acceptance of electric automated vehicles after exposure in a realistic traffic environment

**DOI:** 10.1371/journal.pone.0215969

**Published:** 2019-05-02

**Authors:** Jan C. Zoellick, Adelheid Kuhlmey, Liane Schenk, Daniel Schindel, Stefan Blüher

**Affiliations:** Charité – Universitätsmedizin Berlin, corporate member of Freie Universität Berlin, Humboldt-Universität zu Berlin, and Berlin Institute of Health, Institute of Medical Sociology and Rehabilitation Science, Berlin, Germany; Institute of Psychology, Chinese Academy of Sciences, CHINA

## Abstract

After years of hypothetical surveys and simulator studies, automated vehicles (AVs) are now being tested in realistic traffic environments adding validity to knowledge about their acceptance. We present data from a pilot test with participants (*n* = 125) after experiencing a ride in an electric AV on a large clinic area in Berlin, Germany. As a first contribution, we bridge the gap between missing definitions of key constructs, confusion about their operationalisations, and a rigorous test of their statistical properties and data structure by examining scales on acceptance, trust, perceived safety, intention to use, and—for the first time applied to AVs—the emotions amusement, fear, surprise, and boredom. Tests of reliability and normality were satisfying for almost all constructs (Cronbach’s alphas ≥ .69; six of eight scales normally distributed). The vehicles were accepted (*M* = 1.22; *SD* = 0.70; range -2 to 2), trusted (*M* = 3.29; *SD* = 0.81; range 1 to 5), and perceived as safe (*M* = 3.29; *SD* = 1.03; range 1 to 5). However, factor analyses did not reflect the hypothesised data structure, and validity concerns question the suitability of some constructs for attitude assessment of electric AVs. Our open item for comments added valuable insights in qualitative aspects of user attitudes towards electric AVs regarding driving style, technical features, and (unsettling) audio-visual feedback. We thus argue for broader conceptualisations of key constructs based on interdisciplinary exchange and multi-methodical study designs.

## Introduction

**Fig 1:**
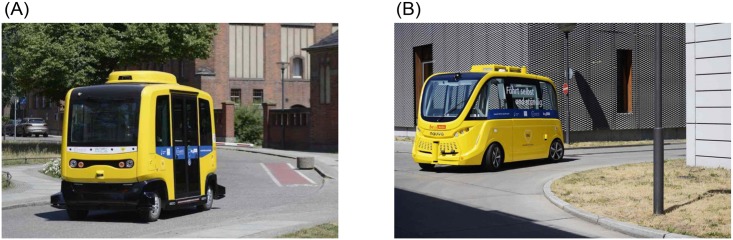
Electric, shared AVs at the Charité campuses. (A) EasyMile EZ10 at Charité Campus Mitte. (B) Navya Arma at Charité Virchow Klinikum. Republished from [15] under a CC BY license, with permission from Charité –Universitätsmedizin Berlin, original copyright 2018.

The development of automated vehicles (AVs) presents a caesura in mobility [1–3] revolutionising travel particularly for people in old age and with disabilities [4, 5]. In this paper, we understand AVs to be shared, electrically powered, and to feature automation above SAE level 4 being able to perform at least “all driving functions under certain conditions” [6]. These vehicles are pod-like, equipped with window fronts on all sides and opposing seats, and exhibit no obvious front and rear setting them apart from both passenger cars and public transport vehicles [7]. Fig 1 presents a picture of the AV used in this study. With these alterations, it is unclear how people will react in encounters as co-habitants or as potential users. Research has identified several attitudes relevant for the user assessment of AVs [7, 8]. However, a variety of definitions and operationalisations [9, 10] combined with hypothetical study designs [11, 12] and rather descriptive analyses [13, 14] lead to uncertainty and confusion about people’s attitudes towards AVs.

With this article, we propose a standardised procedure to approach the topic of AV attitude research including (1) transparency of definitions and operationalisations, (2) availability of all data and questionnaires, (3) rigorous reporting of data structure, reliability, and statistical properties, (4) critically evaluating the own approach, and (5) positioning the findings within the corpus of existing research. This is our major contribution. It supports researchers in the field to improve their instruments. Secondly, we contribute to research methods by demonstrating how open items can add value in an otherwise quantitative survey opening the gate to a deeper understanding of key attitudes.

### Literature review

Several authors have modelled (the relationships between) attitudes towards AVs [7, 8, 16]. They borrowed from the literature on general technology acceptance (e.g., TAM [17, 18] or UTAUT [19]) enriching them with psychological constructs (e.g., locus of control [20] or trust [21, 22]) and mobility focus. The most comprehensive AV attitude models to our knowledge [7, 8] feature outcome variables such as behavioural intentions, usefulness, satisfaction, social acceptability, or willingness to pay and multiple predictors such as socio-demographics, trust, perceived safety, pleasure, and arousal. While some articles on AV attitudes apply and test these models [23, 24], many others develop ad hoc models of their own [25–28] or do not provide models or conceptualisations of their measures at all [13, 14, 29, 30]. Theoretical perspectives on attitudes towards AVs thus have not diffused properly into the discourse. Yet, without adequate models, hypothesis testing and comparisons of effect estimates between studies become difficult and studies remain descriptive. This is a first limitation of the body of work on AV attitudes.

A second limitation emerges from the physical inaccessibility of AVs—inviting people to paint blossoming landscapes of cities without congestion, parking, and accidents in the new era of sustainable mobility [3] with more inclusion particularly for people of old age and with disabilities [5]. Consequently, empirical research has relied on hypothetical scenarios [11, 12] or simulator studies [16, 31] with consequences for the validity of their findings. Having conducted a stated choice experiment with costs and waiting times for automated rides Krueger et al. [12] suspect “a hypothetical bias may be present in the data due to the hypothetical nature of the stated choice experiment, i.e. the results obtained in this study might be of limited value in realistic settings”. Does the willingness to pay US$5,857 for adding level 4 automation [11] translate into behaviour once the system is available? “As […] more technological experiences start spilling into the public domain, these perceptions, and potential behavioral responses are apt to change” [11]. With knowledge about AVs being rather abstract, data from current surveys on attitudes are “possibly of only limited validity, for the object of the survey is not yet clearly defined, as people have hardly encountered it” [32]. Thus, the level of experience made in an experimental setting influences the validity of results [33]. This is important when comparing studies offering actual rides with those offering nothing but a definition of AVs next to a picture.

As a third limitation, studies differ in their definitions of concepts and their operationalisations. For acceptance alone, Adell [9] identified four categories of definitions ranging from attitudes like satisfaction or usefulness to actual system use. Different definitions then lead to different measurement approaches. Accordingly, Adell [9] categorised acceptance measures into eight different groups with 22 sub-groups clearly complicating comparisons between studies. Unfortunately, for other concepts the picture looks similar. Two meta-analyses on trust in human-machine interaction and automation did not even attempt to define the concept—yet both calculated effect sizes for outcomes of trust and their moderators [34, 35]. With uncertainty about trust as a concept, its measurement also varies. Choi and Ji [24] measured trust in AVs with three items retrieved from a study on trust in e-commerce [21], whereas Verberne et al. [31] used twelve “Likert-type items” of unreported origin for their trust measurement. This story of missing definitions and diverse measurements repeats for perceived safety and perceived risk [8, 16, 24].

This diversity in methods, however, creates uncertainty and confusion, unless researchers make the process of data conduction, analysis, and reporting as transparent as possible. In line with Adell et al., we argue for clear study protocols and reporting of methodological approaches and measurements [10]. This is the most promising way to enable comparisons between studies and build a shared basis for understanding peoples’ assessment of AVs. The present study contributes to this base by defining each construct and providing detailed accounts of all measures. It is a pre-registered pilot test [36] with a clear methodological focus (registration link https://osf.io/92pv5/).

## Methods

The Charité data protection bureau (written vote 598/17/ST3) and the Charité ethics committee (written vote EA2/188/17) have approved of the study. The data security bureau and ethics committee waived the need for written consent, because obtaining written consent would have rescinded anonymity. Therefore, we only obtained verbal consent with parents/guardians verbally consenting for minors. Consent was informed based on an information sheet clarifying the topics of voluntariness, anonymity, and data processing for scientific purposes.

### Definitions

Based on theoretical models for AV attitudes [7, 8], we investigated the following latent constructs: acceptance, perceived safety, intention to use, trust, and the emotions amusement, fear, surprise, and boredom. Those attitudes are employed most commonly in the literature and—as part of the models—can be used for hypothesis testing following this pilot study. We decided against constructs from other models (e.g., *perceived ease of use* from TAM [17] or *effort expectancy* from UTAUT [19]), because they cannot be applied easily to automated technology. Table 1 provides the definitions of all eight constructs used in this study. Note that any emotion depicts a “complex phenomenon having neurophysiological, motor-expressive, and experiential components” [37]. We only assess the experiential component. We advise particular caution regarding acceptance as researchers define this concept very differently [10]. In fact, Adell proposes acceptance to be “the degree to which an individual intends to use a system and, when available, to incorporate the system in his/her driving” [9]. For comparisons of our results, it is important to note that her acceptance measure corresponds to our intention to use measure, *not our acceptance measure*. We chose particularly concepts close to experience rather than imagination. Willingness to pay, for example, assumes availability, pricing mechanisms, and scenarios of possible usage, whereas intention to use only assumes the latter.

**Table 1:** Definitions of the eight latent constructs applied in the pilot-study.

Concept	Definition
Acceptance	Direct attitudes towards a system, i.e. predispositions to respond, or tendencies in terms of ‘approach/avoidance’ or ‘favourable/unfavourable’ [38]
Perceived safety	A subjective evaluation of the hazard for the physical condition of the passenger both generally and with consideration of attention/distraction [8]
Trust	The belief that allows users to willingly become vulnerable to automated vehicles after having considered its characteristics [21]
Intention to use	A person’s location on a subjective probability dimension involving a relation between oneself and taking a ride in an automated vehicle [39]
Amusement	The conscious experience of positive valence and high arousal belonging as a shade to the emotional family of joy [37, 40]
Fear	The conscious experience of negative valence and high arousal related to but more activating than distress with a high potential to trigger behavioural responses of ‘fight or flight’ [37]
Surprise	The conscious experience of high arousal triggered by misexpected (positive or negative) stimuli resulting in a short-lasting impetus for behaviour [37]
Boredom	The conscious experience of slightly negative valence and low arousal resulting from indifference and languidness [37, 40]

### Measures

Our two-paged pilot test questionnaire contained 36 items. The used German questionnaire (S1 Appendix) as well as an English version (S2 Appendix) can be found in the supplementary material. Next to questions regarding age, gender, driver’s license, and an open item for comments on the project and the vehicles, we measured the following latent constructs:

- *Acceptance of AV* measured using the “simple procedure”–a five-point semantic differential from colleagues [38]
- *Perceived safety* measured using four out of six items from colleagues [8]. We exclude two items beforehand, because they could not be adapted to AVs in a sensible way.
- *Trust* measured using three adapted items from colleagues [21] to fit the context of AVs [24]
- *Intention to use* measured using three items from colleagues [8]
- *Emotions* associated with AVs (particularly *surprise* and *fear*) measured using the DAS (the German translation of the DES [41]) by colleagues [42] and *boredom* and *amusement* using the M-DAS (the modified version of the DAS) by colleagues [43]

For each word pair of the acceptance measure, we used a scale from +2 to -2, reversing items 3, 6, and 8 as proposed by the authors [38]. For measuring perceived safety, intention to use, and trust we used a 5-point Likert scale with the range *disagree*, *somewhat disagree*, *neither agree nor disagree*, *somewhat agree*, and *agree*. We coded the scale from 1 (*disagree*) to 5 (*agree*). Items 1 and 2 from the perceived safety scale were reversed.

For the emotions measures in the pilot test, we deviated from the AsPredicted Preregistration in two ways. First, we used the emotion amusement from the M-DAS [43] instead of joy from the original DAS [42]. This was because in Renaud and Unz’s [43] two reliability studies, amusement had Cronbach’s alphas of.90 and.86 compared to.87 and.88 of joy. We wanted to see, whether we could replicate findings for this less well-researched emotion and compare our findings to those of other studies. The four emotions represented different levels of valence (amusement and fear) and different levels of activation (boredom and surprise) [40]. Second, we used a five-point Likert scale ranging from *very weak* to *very strong* answering the question “How did you feel on the ride with the electric automated bus? Please give your evaluation for the following terms”. This deviates from the applications of colleagues [43, 44] who used an asymmetric scale ranging from *not at all* to *very much* answering the questions “I felt… angry” or “I experienced… joy”. We opted for the Likert scale because of assumed equidistance reaching interval scale levels instead of ordinal levels in asymmetric scales enabling us perform parametric calculations. This also aligns with guidelines for scale construction by colleagues [45].

Lastly, we measured general perceived safety with the question “How safe did you feel on the ride with the electric automated bus?” on a five-point Likert scale with the range very unsafe, unsafe, neutral, safe, and very safe. We coded the scale from 1 (*very unsafe*) to 5 (*very safe*).

### Procedure

In accordance with our pre-registration (registration link https://osf.io/92pv5/), we performed a pilot test investigating latent constructs during the so-called Long Night of the Sciences at the Charité campuses Virchow-Klinikum (CVK) and Mitte (CCM) on 9 June 2018.

**Fig 2:**
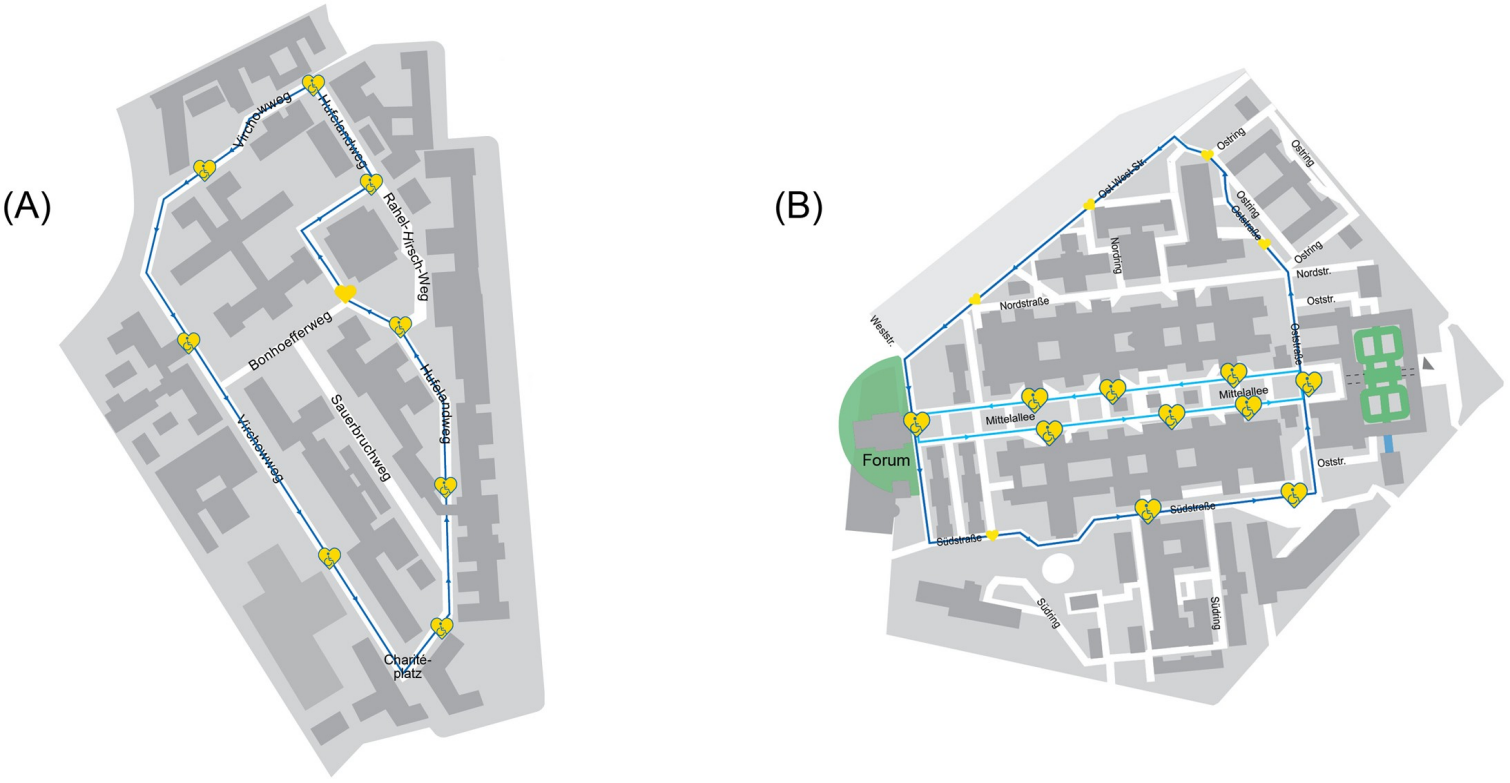
Maps of the campus areas with AV routes marked in blue. Hop-on-hop-off stations depicted as yellow hearts; wheelchairs symbolise stations equipped for the disabled. (A) Charité Campus Mitte with AVs driving counter clockwise. (B) Charité Virchow Klinikum with AVs driving counter clockwise; route of the AVs used in the pilot study in light blue; route additionally used in the project in dark blue. Republished from [46] under a CC BY license, with permission from Charité –Universitätsmedizin Berlin, original copyright 2017.

The Long Night of the Sciences is a special event with more than 70 participating universities, museums, and other institutions in Berlin and Potsdam providing knowledge and entertainment for more than 28.000 visitors (https://www.langenachtderwissenschaften.de/index.php?article_id=534). Visitors could also take a ride with one automated vehicle (AV) along a round course on each campus. Even though situated on private land with a speed limit of 20 km/h, both courses were set in a realistic environment with asphalt grounding, intersections, the necessity to perform turns, occupied parking spaces and greening at the road sides, pedestrian crossings, and pedestrians, cars, and cyclists as road users. The course at CCM additionally exhibited a level crossing communicating with the AV via radio-frequency identification (RFID). The course at CVK was 0.85 km long and had eight hop-on-hop-off stations; the course at CCM was 1.20 km long and had nine hop-on-hop-off stations. One round lasted 10 to 15 minutes. Fig 2 presents the campuses with highlighted round courses. The Navya Arma drove at CVK, and the Easymile EZ10 drove at CCM. Both AVs were electrically powered, navigated through LIDAR sensors and GPS signals, and had a maximum speed of 12 km/h. They performed all driving functions (e.g., accelerating, braking, or opening doors) automatically alongside the programmed routes. A so-called operator, i.e. a person able to navigate the vehicle with a remote control, supported each AV at all times. The operator also provided information on the technology and the project. Project partners included the Berliner Verkehrsbetriebe (BVG) and the Berlin Senate Department for the Environment, Transport and Climate Protection.

We approached people exiting the AVs at the two most frequently used stations on each campus inviting them to participate in a short questionnaire study without codified criteria who to ask. After explaining the conditions for participation (e.g., voluntariness, anonymity, and data processing for scientific purposes all presented on an information sheet), we handed them a clipboard with the two-page questionnaire. Participants received no material compensation.

### Sample

535 people took a ride with an AV. Of those, 125 people participated in our pilot-study (23% participation rate). With four data collectors and seventeen hop-off stations on both campuses combined, we were unable to ask every passenger for their participation in our study. We did not use codified criteria which passenger to approach and did not exclude anyone from participation. 62 participants were male. On average, participants were 33.00 years old (*SD* = 16.35). 69% had a driver’s license. Of those without a driver’s license, 56% were under 17 years old, i.e. the age to obtain a license in Germany. 61% participated at CCM and 39% at CVK. Table 2 shows sample characteristics for both campuses.

**Table 2:** Sample characteristics split between campuses.

	CCM	CVK
Participants, *n* (%)	76 (61%)	49 (39%)
Age in years, *M* (*SD*)	36.45 (17.23)	28.12 (12.90)
Male participants, *n* (%)	41 (54%)	21 (43%)
Underage participants, *n* (%)	12 (16%)	11 (22%)
Participants without driver’s license, *n* (%)	21 (27%)	18 (37%)

*N* = 125; CCM, Charité Campus Mitte; CVK, Charité Virchow Klinikum.

### Analysis

Before imputing data, it is necessary to check the missing data distribution. Imputing data missing not at random produces biased estimates [47]. Little’s MCAR test is a first step that analyses if data is missing completely at random (MCAR). If significant, the MCAR hypothesis has to be rejected and further investigation is necessary to explain the structure behind missing data. Unfortunately, to our knowledge there is no test for checking if data is missing at random or not at random. Assuming one or the other with probable cause and estimating the introduced bias seems to be the only option [47]. Accordingly, we performed Little’s MCAR test using SPSS 25.0 [48] on the raw data to understand the structure of missing data. Our data set contained 3.44% missing values. Little’s MCAR test was significant (χ^2^ = 573.164, df = 496, p = .009) meaning data were not missing completely at random. 6 out of 36 items had more than 5% missing values. They belonged to the constructs acceptance (2), trust (2), perceived safety (1), and the emotion fear (1). Further investigations revealed that four participants (0% men, *M* = 29.75 years, *SD* = 19.72 years, 50% driver’s license) accounted for 55% of all missing values—most of them answered no item on the entire second page, presumably because they did not turn the paper. Re-calculating Little’s MCAR test with those participants excluded who did not answer the second page at all, we still obtained significant results (*χ*^*2*^ = 532.950, *df* = 470, *p* = .023). However, no item had more than 5% missing. We thus assumed data to be missing at random and imputed missing values using the expectation maximisation (EM) algorithm in SPSS version 25.0. EM estimates missing values based on a linear regression with all other data as predictors [47]. Thus, all imputed data using EM lie on a linear graph narrowing the data’s variance. Nonetheless, this this procedure is suggested with less than 5% missing values [47]. We used naïve rounding for gender, but refrained from further use leaving imputed data as they are even if they did not fit into answer categories of the (Likert) scales [49]. We did not exclude any quantitative data.

To understand the data structure, we performed an exploratory factor analysis (EFA) with principal component extraction on all constructs simultaneously as a measure of construct validity. We applied oblique rotation (direct oblimin) and set an Eigenvalue of 1 and visual examination of the scree plot as criteria for extraction [36, 50]. We expected to find nine factors representing the nine latent constructs (acceptance being separated into the two subscales usefulness and satisfying). We also modelled two confirmatory factor analyses (CFAs) in SPSS Amos using expectation maximisation with items loading on the corresponding variable and correlations between all latent variables. The first CFA split acceptance in its subscales usefulness and satisfying whereas the second CFA had acceptance as one latent variable with all items loading. A visualisation of the first model (S1 Model) can be found in the supplementary material. For interpretation we applied cut-offs proposed by colleagues [51]. With that, we could identify whether acceptance, perceived safety, intention to use, trust, and the four emotions are indeed separate constructs.

We then analysed each scale individually calculating EFAs, CFAs (where applicable), Cronbach’s alphas as reliability measures, item statistics (difficulty and item-scale correlations) and descriptive statistics. We considered item-scale correlations *r*_*it*_ > .50 as high and difficulties between.20 and.80 as satisfying [52]. We applied skew and excess kurtosis measures as indicators for normal distribution. Garson [53] suggests normality if both skewness and excess kurtosis fall in the range between +2 and -2. Lastly, we calculated means for all scales and tested if they differed significantly from the neutral middle of the scale using t-tests with Bonferroni-corrected alpha levels (α = .05 / 7 = .007).

We analysed data from the open comment item based on Kuckartz’s [54] qualitative content analysis. Using MAXQDA version 11.0 [55], two members of our research team (both male, *M* = 23.5 years, one with prior experience using the method and software, the other trained for this project) independently formed categories inductively and consolidated them based on consensus. Two other team members (both female, *M* = 22.5 years, one with prior experience using the method and software, the other trained for this project) independently assigned the comments to the identified categories using one-to-many classification. We calculated agreement percentages and fuzzy kappa as a measure of interrater reliability [56]. The two coders discussed conflicting tags in one meeting and recoded the material.

## Results

### Data structure

We expected to find nine factors in our EFA representing the scales usefulness (acceptance subscale), satisfying (acceptance subscale), trust, perceived safety, intention to use, amusement, fear, surprise, and boredom. However, only seven factors presented Eigenvalues ≥ 1 (namely 8.72, 3.86, 2.85, 1.87, 1.59, 1.41, and 1.16) explaining 28%, 12%, 9%, 6%, 5%, 4%, and 3% of variance, respectively. Factor loadings can be found in the supplementary material (Table A in S1 Text). Items from the scales for acceptance (factor 1), fear (factor 3), and surprise (factor 6) loaded on respective factors without crossloadings. All items for trust (factor 4), intention to use (factor 5) and amusement (factor 2) loaded on one factor, respectively. However, all three had crossloadings with items from other scales. These crossloadings came from the scales perceived safety (factors 4 and 5) and boredom (factor 2). Factor 7 included two items from boredom. This data structure suggests that perceived safety was difficult to distinguish from other concepts (namely trust and intention to use), and that the boredom scale did not create a uniform construct. Additionally, we were unable to replicate the two-factor solution for acceptance proposed by [38]. In contrast to the Eigenvalue criterion, the scree plot suggested retrieving three additional factors with Eigenvalues of 0.93, 0.85, and 0.82 (Fig in S1 Text). In this 10-factor solution items from the scales for amusement (factor 2), fear (factor 3), trust (factor 5), and surprise (factor 6) formed unique factors. Only six items from the acceptance scale loaded on factor 1 while the other three items loaded weakly on multiple factors. The scales for boredom and perceived safety were split across multiple factors as in the 7-factor solution. Factor 9 had no item with an acceptable factor score [36], but multiple crossloadings ≥.32 with other factors [57] from items belonging to three different scales. The pattern matrix can be found in the supplementary material (Table B in S1 Text). However, factors with Eigenvalues < 1 yield less explanatory power than a single item questioning the usefulness of retrieving them instead of using the item for further analysis [58]. Thus, the 10-factor solution did not provide additional information of practical use for further analysis.

Our first CFA (n = 125, no missing data) with nine correlated latent variables (acceptance split into usefulness and satisfying) resulted in poor model fits (χ^2^ = 767.173, df = 398, *p* < .001; CFI = .82; TLI = .80; and RMSEA = .09). Model fits did not increase when we combined the two acceptance sub-scales to one latent variable. This means acceptance was not the main reason for poor model fit.

In summary, the hypothesised data structure did not adequately represent the empirical data—supposedly because of the scales perceived safety and boredom found in the EFA to load unexpectedly. The next sections provide further insights into each individual scale to explore their characteristics and quality.

### Measure statistics

#### Acceptance

Van der Laan et al. propose their scale to result in a two-factor structure with the items 1, 3, 5, 7, and 9 loading on the first factor named *usefulness*, and the items 2, 4, 6, and 8 loading on the second factor named *satisfying* [38].

The EFA with oblique rotation resulted in one factor with an Eigenvalue of 5.29 explaining 59% of variance. The factor loadings for every item are listed in Table 3. The scree plot clearly suggested a single-factor solution (S1 Fig).

**Table 3:** Pattern matrix with oblique rotation for the acceptance scale.

Item	Factor 1
Useful—useless	.84
Pleasant—unpleasant	.83
Bad—good	.81
Undesirable—desirable	.80
Assisting—worthless	.78
Effective—superfluous	.78
Irritating—likeable	.75
Nice—annoying	.72
Raising alertness—sleep-inducing	.57

Items bad—good, irritating—likeable, and undesirable—desirable were reversed.

In our CFA (n = 125, no missing data), we first modelled two correlated latent variables (*usefulness* and *satisfying*) as described above resulting in poor model fits (χ^2^ = 75.800, df = 26, *p* < .001; CFI = .92; TLI = .89; and RMSEA = .12). We then modelled one single latent variable with all items loading resulting in similarly poor model fits (χ^2^ = 81.684, df = 27, *p* < .001; CFI = .91; TLI = .88; and RMSEA = .13). Thus, we were unable to find the two-factor structure proposed by colleagues [38] in both factor analyses.

Reliability analyses for the two sub-scales revealed Cronbach’s alphas of.84 for usefulness and.84 for satisfying. Cronbach’s alpha for the entire scale was.91 –unsurprisingly larger than for the subscales, because more items were included in the calculation. Descriptive statistics of each item are displayed in Table 4. Van der Laan et al. [38] and many applicants of the scale assume interval scale level enabling the calculation of means [59, 60]. For those, we are reporting mean and standard deviation. However, this might result in overestimation of significance since “[v]iolations of data level assumptions mean that actual standard error will be greater than the computed standard error” [53]. For those convinced of the scale’s ordinal nature we also report the median. Items 1 and 7 exceeded the range of excess kurtosis. All other items were normally distributed. The difficulties of items 1, 2, 3, 6, 7, and 8 exceeded the preferable range [52], meaning that they most participants answered with high values. All item-scale correlations except for item 9 were high. Contrary to Van der Laan et al. [38] we have found their acceptance scale to be a narrow construct [52].

**Table 4:** Descriptive statistics of the acceptance scale.

Item	Median	Mean (SE)	SD	Skew (SE)	KU (SE)	Difficulty	Item-scale correlation
Useful—useless	2.00	1.37 (0.08)	0.92	-1.84 (0.22)	3.54 (0.43)	.84	.77
Pleasant—unpleasant	1.00	1.18 (0.08)	0.93	-1.05 (0.22)	0.52 (0.43)	.80	.76
Bad—good	1.00	1.24 (0.08)	0.84	-0.90 (0.22)	0.55 (0.43)	.81	.74
Nice—annoying	1.00	1.09 (0.08)	0.91	-0.78 (0.22)	0.08 (0.43)	.77	.65
Effective—superfluous	1.00	1.10 (0.09)	1.02	-1.12 (0.22)	1.13 (0.43)	.78	.70
Irritating—likeable	1.54	1.33 (0.07)	0.78	-1.00 (0.22)	0.37 (0.43)	.83	.67
Assisting—worthless	2.00	1.27 (0.09)	0.99	-1.57 (0.22)	2.29 (0.43)	.82	.72
Undesirable—desirable	1.00	1.23 (0.08)	0.90	-1.32 (0.22)	1.84 (0.43)	.81	.72
Raising alertness—sleep-inducing	1.00	0.81 (0.09)	1.04	-0.53 (0.22)	-0.35 (0.43)	.70	.49

*N* = 125; SE, standard error; SD, standard deviation; KU, excess kurtosis; scale range -2 to 2; items 3, 6, and 8 were recoded.

In summary, we could not replicate the two-factor structure of the acceptance scale and thus support the one-factor solution reported by colleagues [61, 62]. Acceptance seems to a narrow construct. Reliability of the scale was satisfying. Agreement with all items was high resulting in large and positive acceptance for electric AVs.

#### Perceived safety

The EFA with oblique rotation resulted in one factor with an Eigenvalue of 2.02 explaining 73% of variance. Factor loadings for items 1, 2, 3, and 4 were.83,.77,.68, and.53, respectively. The scree plot indicated a single-factor solution (Fig A in S2 Text). For further exploratory purposes, we added our self-constructed item for general safety to the EFA and extracted two factors with Eigenvalues of 2.17 and 1.10 explaining 43% and 22% of variance, respectively. The scree plot suggested a two-factor solution. Factor loadings (Table in S2 Text) and scree plot (Fig B in S2 Text) can be found in the supplementary material. In our CFA, we modelled one single latent variable with all four items loading and uncorrelated variances. This resulted in an acceptable model fit (χ^2^ = 1.445, df = 2, *p* = .49, TLI = 1.02, CFI = 1.00, and RMSEA = .00).

Reliability analysis resulted in a Cronbach’s alpha of.64. The reliability increased to.69, if we dropped item 2. Adding our self-constructed fifth item did not increase Cronbach’s alpha above.64. In line with our EFA and reliability results, we dropped item 2. We recalculated the EFA with oblique rotation and three items resulting in one factor with an Eigenvalue of 1.86 explaining 62% of variance. Descriptive statistics of the perceived safety scale can be found in Table 5.

**Table 5:** Descriptive statistics of the perceived safety scale and self-constructed Item.

Item	Median	Mean (SE)	SD	Skew (SE)	KU (SE)	Difficulty	Item-scale correlation
*I believe using AVs is dangerous*. (item 1)	4.00	3.90 (.08)	0.92	-0.44 (.22)	-0.36 (.43)	.73	.58
*Using AVs requires increased attention*. (item 2)	3.00	3.22 (.12)	1.28	-0.28 (.22)	-0.97 (.43)	.56	.29
*I feel safe when using AVs*. (item 3)	4.00	3.64 (.10)	1.06	-0.34 (.22)	-0.43 (.43)	.66	.38
*Using AVs decreases the accident risk*. (item 4)	3.00	3.51 (.09)	1.01	0.02 (.22)	-0.32 (43)	.63	.49
*How safe did you feel on the ride*? (self-constructed)	4.00	4.42 (.08)	0.89	-1.40 (.22)	2.50 (.43)	.81	–

*N* = 125; SE, standard error; SD, standard deviation; KU, excess kurtosis; scale range 1 (*disagree*) to 5 (*agree*). Items 1 and 2 are reversed. Item 2 was dropped from further analyses.

Data for all items on the perceived safety scale was normally distributed. Difficulty for all scale items was satisfying. Item-scale correlations for items 2 and 3 was rather low. Our self-constructed item displayed an excess kurtosis outside the boundaries of assumed normality. Its difficulty was outside the satisfying range meaning that participants were too inclined to answer affirmatively.

In summary, the perceived safety scale did not perform as expected since item 2 was weakly connected to the rest of the scale. Focusing on attention rather than safety this disconnect is also understandable regarding the content of the items. After excluding item 2, we interpreted the factor as ‘perceived safety’. The internal consistency was on the lower boundary of acceptability. Thus, perceived safety might be a broader concept, particularly compared to the others in this article. Our self-constructed item was impractical for further statistical analyses since it was non-normally distributed, easy (difficulty > .80), and weakly connected with the scale supposedly measuring something similar. We thus cannot promote this self-constructed item for statistical analyses.

#### Intention to use

The EFA with oblique rotation resulted in one factor with an Eigenvalue of 2.26 explaining 75% of variance. The factor loadings for the items 1, 2, and 3 were 0.85, 0.89, and 0.86, respectively. The scree plot suggested a single-factor solution (S2 Fig). Reliability analysis resulted in Cronbach’s alpha of.83. Descriptive statistics of the intention to use scale can be found in Table 6. Data on the intention to use scale was normally distributed. The difficulty was within satisfying range for all items and all item-scale correlations were high.

**Table 6:** Descriptive statistics of the intention to use scale.

Item	Median	Mean (SE)	SD	Skew (SE)	KU (SE)	Difficulty	Item-scale correlation
*Assuming I had access to an AV*, *I intend to use it* (item 1)	4.00	3.96 (.10)	1.12	-0.77 (.22)	-0.54 (.43)	.74	.67
*Given I had access to AV*, *I predict that I would use it* *(item 2)*	4.00	3.66 (.11)	1.20	-0.65 (.22)	-0.50 (.43)	.66	.73
*If AVs are available*, *I plan to use one in the next months* *(item 3)*	3.72	3.42 (.12)	1.32	-0.32 (.22)	-1.06 (.43)	.60	.69

*N* = 125; SE, standard error; SD, standard deviation; KU, excess kurtosis; scale range 1 (*disagree*) to 5 (*agree*).

In summary, the intention to use scale produces a narrow construct with one factor in the EFA and high Cronbach’s alpha and item-scale correlations. Being normally distributed and reliable, we consider it adequate for further analyses.

#### Trust

We expected all three items measuring trust to load on a single factor. The EFA with oblique rotation resulted in one factor with an Eigenvalue of 2.08 explaining 69% of variance. The factor loadings for items 1, 2, and 3 were 0.86, 0.82, and 0.82, respectively. The scree plot suggested a single-factor solution (S3 Fig).

Reliability analysis of the scale resulted in Cronbach’s alpha of.77. Descriptive statistics of the trust scale can be found in Table 7. Data on the intention to use scale was normally distributed. The difficulty of all items was within satisfying range and all item-scale correlations were high.

**Table 7:** Descriptive statistics of the trust scale.

Item	Median	Mean (SE)	SD	Skew (SE)	KU (SE)	Difficulty	Item-scale correlation
*AVs are trustworthy*. (item 1)	3.00	3.40 (.08)	0.88	-0.19 (.22)	0.25 (.43)	.60	.65
*AVs keep promises and commitments*. *(item 2)*	3.00	3.25 (.08)	0.89	-0.03 (.22)	0.29 (.43)	.56	.59
*I trust AVs*, *because they keep my best interests in mind*. *(item 3)*	3.00	3.21 (.10)	1.15	-0.31 (.22)	-0.28 (.43)	.55	.59

*N* = 125; SE, standard error; SD, standard deviation; KU, excess kurtosis; scale range 1 (*disagree*) to 5 (*agree*).

In summary, the trust scale produces a narrow construct with one factor in the EFA and high Cronbach’s alpha and item-scale correlations. Being normally distributed and reliable, we consider it adequate for further analyses.

#### Emotions

We expected four factors each with three items loading exclusively on the respective factor. The EFA with oblique rotation resulted in three factors Eigenvalues of 3.50, 2.80, 1.41, explaining 29%, 23%, and 12% of variance, respectively. The pattern matrix can be found in the supplementary material (Table A in S3 Text). The first factor had loadings of amusement (positive) and boredom (negative), and the other two represented fear and surprise. However, the scree plot (Fig in S3 Text) suggested a five-factor solution. The additional factors had Eigenvalues of 0.95 and 0.90 explaining 8% and 7% of variance, respectively. We re-calculated the EFA with a lower Eigenvalue threshold to explore the two additional factors. Factor loadings of the five factors can be found in the supplementary material (Table B in S3 Text). The first three factors represented amusement, fear, and negative surprise. The boredom items split between factors four (“bored” and “bored stiff”) and five (“uninvolved”).

Reliability analyses of the proposed emotions resulted in Cronbach’s alphas of.77 (amusement),.86 (surprise),.81 (fear), and.63 (boredom). Because of reliability concerns and the results from the EFAs, we dropped the boredom scale and re-calculated the EFA with oblique rotation. We extracted three factors with Eigenvalues of 2.97, 2.31, and 1.37 explaining 33%, 26%, and 15% of variance, respectively. Their factor loadings are displayed in Table 8.

**Table 8:** Pattern matrix with oblique rotation for the emotions surprise, fear, and amusement.

Item	Factor 1	Factor 2	Factor 3
Amazed	**0.93**	0.08	0.12
Astonished	**0.87**	0.16	0.23
Surprised	**0.81**	0.11	0.02
Fearful	0.06	**0.90**	-0.03
Scared	0.12	**0.82**	0.01
Afraid	0.14	**0.81**	-0.10
Amused	0.07	-0.04	**0.85**
Silly	0.15	-0.12	**0.81**
Fun-loving	0.09	0.04	**0.80**

Item loadings with an absolute value above.50 are displayed in bold.

We calculated sum scores for amusement, surprise and fear by adding the responses for the respective items resulting in a scale range from 3 to 15. Descriptive statistics of amusement, surprise, and fear can be found in Table 9. The scales for amusement and surprise were normally distributed. However, fear was highly skewed and spiky. Results from t-tests indicate that participants interpreted the electric AVs as amusing, surprising, and not fear inducing.

**Table 9:** Descriptive statistics of the emotions scale.

Variable	Median	Mean (SE)	SD	Skew (SE)	KU (SE)	t-Value
Amusement	12.00	11.66 (.20)	2.24	-0.43 (.21)	-0.06 (.43)	13.31**
Surprise	10.00	9.86 (.28)	3.08	-0.16 (.21)	-0.54 (.43)	3.12*
Fear	3.00	3.87 (.15)	1.72	2.39 (.21)	6.87 (.43)	-33.40**

*N* = 125; SE, standard error; SD, standard deviation; KU, excess kurtosis; scale range 3 (*very weak*) to 15 (*very strong*); α = .007 (Bonferroni-corrected).

*p < .007;

**p < .001.

In summary, we were unable to retrieve the four emotions as expected. Particularly the boredom scale was internally inconsistent and did not produce a unique factor. We thus cannot recommend the use of this scale in analyses. The other scales produced unique factors with high Cronbach’s alphas suggesting narrow constructs. Amusement and surprise were normally distributed; fear was skewed and spiky. Thus, we recommend caution interpreting this result of fear of AVs in (inferential) statistical analyses. Fear might be too drastic to describe the experience of riding an AV supervised by an operator with 12km/h maximum speed on private land adequately.

#### Summary

All attitude scales were normally distributed and they produced satisfying Cronbach’s alphas comparable to reliability coefficients reported in previous studies. Table 10 provides descriptive statistics of all attitude measures together with analyses of mean deviations from the neutral scale middle. Accordingly, participants accepted and trusted the electric AVs, perceived them as safe, and intended to use one in the future. Similar positive assessments were reported in the emotions measure. Participants were amused, surprised, and not afraid after the ride with an electric AV. Low values on the attitudes and emotions would create a barrier for usage and a disincentive for car manufacturers. Even though marketability of street-legal AVs might still be a decade away [1, 3, 63], our findings are promising for further development of automated urban mobility.

**Table 10:** Descriptive statistics and differences from neutral middle of all constructs.

Item	Median	Mean (SE)	SD	Skew (SE)	KU (SE)	t-Value
Acceptance^a^	1.22	1.18 (0.06)	0.70	-0.96 (0.22)	0.80 (0.43)	18.73**
Perceived safety^b^ (excluding item 2)	3.33	3.29 (0.07)	1.03	-0.16 (0.22)	-0.54	3.12*
Intention to use^b^	4.00	3.68 (0.09)	1.05	-0.47 (0.22)	-0.77	7.22**
Trust^b^	3.27	3.29 (0.07)	0.81	-0.08 (0.22)	0.51	3.97**

*N* = 125; SE, standard error; SD, standard deviation; KU, excess kurtosis; α = .007 (Bonferroni-corrected).

*p < .007;

**p < .001.

^a^scale range -2 to 2.

^b^scale range 1 to 5.

### Comments

30 participants (24%) answered the open comment item “Did we leave something out? Please give us your comments about the project or the vehicles.” Using inductive category formation, two team members independently identified the six categories *AV driving characteristics*, *application scenarios for AVs*, *equipment*, *operator*, *survey method*, and *others*. The category ‘others’ was included to make the list exhaustive and to ensure that each comment was assigned to at least one category. The team members also excluded six comments bereft of much content from further analyses (e.g., “thank you” or “best project”). Two other team members assigned the remaining 24 comments to the six identified categories using one-to-many classification. This resulted in 31 and 33 tags, respectively. Setting segment agreement at 90% both coders assigned the same tag in 84% of cases. This resulted in an interrater reliability of fuzzy kappa = .77. The two coders discussed conflicting tags in one meeting and recoded the material. This resulted in 99% agreement (fuzzy kappa = .97). Table 11 provides an overview of categories and number of entries in each category after conflict resolving.

**Table 11:** Categories and number of entries for answers for the open question.

Category	Number of entries
AV driving characteristics	7.5
Equipment	7
Operator	5
Survey method	5
Other	5
Application scenarios for AVs	4

The half entry (0.5) in AV driving characteristics represents the remaining unresolved conflict of only one coder assigning the category.

According to answers for AV driving characteristics, braking “because of relatively far away targets does not make the ride so pleasant” (M89) and “is very jerky” (F24). One person “cannot evaluate braking” and raised the question “what happens in normal traffic?” (F81). The comments “slow” (M37) and “maiden trip?;-)” (F21) additionally indicated that AVs were not perceived as a valuable addition to mobility services (yet). This stands in contrast to the high median ratings on each intention to use item. No comment lauded AV driving characteristics, but all criticised various facets of the AVs’ behaviours.

For equipment, a major topic was temperature in the EasyMile AVs. Three participants complained about its coldness, but none raised the topic at CVK where the Navya AV drove. Other limitations included hard seats (“more comfortable seats would be preferable” (M73)), loud sounds (“warning signals are too loud when ‘heard’ daily at work” (F98)), and limited sight (“windows in manual mode do not ensure the best view” (F3)). One participant presumably wanted a “tele at the ceiling” (F60).

Participants perceived the two operators differently. The only comment at CVK–“more telling during the ride” (M39)–indicated that the operator should be more talkative. In contrast, one participant at CCM–“assessing the ride today, the operator was a decisive factor—she gabbed a lot though much moonshine” (F77)–found *this* operator to be too talkative. Two other comments lauded the CCM operator as “very, very good” (M107) and the “major reason” for the “grand driving experience” (F112). A last participant stated disappointedly “it’s a shame that an operator is necessary” (F117). These comments underscore the impact of an operator in AV assessment. They might explain to some extent the differences found in AV acceptance and intention to use between the two campuses.

Comments about the survey method underscored our own validity concerns about AV attitude research. One participant identified the operator as a confounding variable limiting the validity of AV assessment (F77). Another participant wished for “I don’t know as a possible choice” (F25) indicating that some (hypothetical) questions about AVs might not be answerable. This missing knowledge base for accurately assessing AVs was the topic of another comment–“I have to gain more passenger car experience with automated technology” (M26). These comments indicate that some participants have difficulties to assess their own attitudes accurately—even after having experienced AVs directly. This questions the informative value of hypothetical surveys even further. A last participant missed a question about her motivation to ride an AV (F36). We will add this question for the following main measurement point.

Scenarios for AV applications differed. One participant “would only drive on private land” (F72), whereas another could not wait for licenses for public roads–“because the legislator unfortunately has not agreed, I hope that it is possible to make on public land as well” (F92). A third participant assessed AVs “for the campus ideal” (M38). Participants did not raise often-cited application scenarios of urban vs. rural areas or shared vs. privately owned AVs by themselves.

The category ‘other’ included comments with various topical foci. Most of them were assigned to another category, i.e. “maiden trip?;-)” (F21) to *AV driving characteristics*; “this was a grand driving experience and the operator was the major reason for this” (F112) to *operator;* “I have to gain more passenger car experience with automated technology” (M26) to *survey method*; and “because the legislator unfortunately has not agreed, I hope that it is possible to make on public land as well” (F92) to *application scenarios*. Lastly, one comment was assigned only to the ‘other’ category demanding “it should be faster! (the development generally!)” (M124).

## Discussion

With our analyses, we tested various statistical properties (e.g., normality and reliability) of multiple scales regularly used to assess attitudes towards automated vehicles. These include acceptance, perceived safety, trust, and intention to use as well as four emotions with differing levels of valence and activation. This pilot test was necessary, because AV attitude research operates with a variety of definitions and measures predominantly in hypothetical study designs focusing on rather descriptive analyses. These factors lead to uncertainty and confusion about people’s attitudes towards AVs with inconsistent results. With all participants having experienced a ride in an AV directly prior answering our survey, our design differed from that of previous studies [12, 25, 32]. Given the direct experience with AVs in our study, we expect our data to have higher validity than designs with hypothetical scenarios and simulated rides.

Our main results include positive evaluations of electric AVs by 125 participants evident in high ratings for acceptance, perceived safety, trust, and intention to use. Participants were amused, surprised, and not afraid after their experience. These results stand in contrast to critical comments addressing uncomfortable interior, slow driving combined with abrupt braking, or the operator as a polarising figure. Our major contribution to the AV attitude research is a template of reporting that includes (1) transparency of definitions and operationalisations, (2) availability of all data and questionnaires, (3) rigorous reporting of data structure, reliability, and statistical properties, (4) critically evaluating the own approach, and (5) positioning the findings within the corpus of existing research. In this section, we focus on the last two points.

With a promising design regarding validity, we found mixed results. Poor model fits in our CFAs indicate that the hypothesised structure is not a good approximation of the data. More nuanced EFA results suggest that some constructs represent the data quite well. Particularly items from the acceptance, surprise, fear, intention to use, and trust scale loaded on individual factors as predicted. Acceptance, perceived safety, trust, and intention to use were normally distributed and exhibited satisfying Cronbach’s alphas (only perceived safety was slightly low with.69) comparable to reliability coefficients reported in previous studies (see pre-registration).

### Critical evaluation and reflexions

However, some difficulties cast a cloud over ingenuous applications of the scales. Statistically, we were unable to replicate the two-factor solution for the acceptance scale—an assumption most applications of the scale do not test [59, 60, 64]. Yet more worrisome are validity concerns. As a semantic differential, the acceptance scale consists of opposites as broad and basic as good—bad. Their informative value about the specific applications might therefore be quite low. Some pairs, such as nice—annoying or assisting—worthless, are arguably not even opposites. This calls into question at least the interval level of the scale if not the interpretation of any given answer.

Similar concerns exist for the other scales. The intention to use scale consists of three almost identical items easily reaching the highest Cronbach’s alpha with the fewest items. However, applied to AVs as an emerging technology all of them linger in the realm of future possibilities. Even though the case at the Charité campuses provides an application scenario, the wording of the items remains vague questioning the validity of the findings. The case of vagueness applies also to the trust measure. “My best interests” that the AV supposedly keeps in mind are not specified—nor are the “promises and commitments” it makes. These wordings make sense in their development context, namely assessing consumer trust in salespeople who have their own interests and make (potentially exaggerated) promises about their commodities [22]. However, particularly inexperienced laypeople might not be able to assess accurately any promises, commitments, or interests of the AVs.

These findings are particularly troublesome, since they are widely used in AV attitude research. With reliability and validity concerns in an arguably more promising design, our findings question the knowledge produced by AV attitude research so far. Further replication, validations, and refinement of scales is needed to assess the appropriateness of these measures and the quality of knowledge in the discourse. We hope, our approach to the topic supports other researchers in their research projects.

Several options exist for overcoming these limitations, e.g. the use of different scales. Instead of defining and operationalising acceptance following Van der Laan et al. [38], researchers could use *performance expectancy* from the Unified Theory of Acceptance and Use of Technology (UTAUT) [19] or *perceived usefulness* from the Technology Acceptance Model (TAM) [17]. However, this means ‘losing’ a satisfaction dimension present in [38] limiting compatibility of results. In addition, UTAUT and TAM have been developed in a labour-related context with particular interpretations of performance and productivity. It is questionable whether they could be adopted easily to users of automated technology. Substitutes for trust and perceived safety might be anxiety [8, 16, 19] and perceived risk [21, 24], respectively, as they can be interpreted as polar opposites. An option for handling duplications (e.g., in the case of intention to use) might be to eliminate items. However, this approach reduces Cronbach’s alphas resulting in potentially unreliable scales. For example, Cronbach’s alpha of our 3-item perceived safety scale (.69) was smaller than the.86 from Osswald et al.’s [8] 6-item scale.

The emotions measures did not work as anticipated. First, we only extracted three unique factors with *boredom* as the most difficult case. Given low reliability ratings in the present study and previous literature [43], we recommend not to use the M-DAS for the measurement of *boredom*. Second, participants independently expressed humour and surprise about the entire scale. Particularly, the German term for “bored stiff” is rather archaic and—as in English—difficult to distinguish from “bored”. Some participants remarked the perceived repetition of terms similar to the intention to use items. Third, particularly the emotion fear might be unfit for our research context. Both AVs drove at maximum speed of 12 km/h on private land with a trained operator on board making the experience of fear quite unlikely—represented by the highly skewed and spiky distribution of fear values. This scenario does not resemble recent deadly accidents with high automation criticised in the media [65]. Thus, conducting more subtle emotions of the same family, e.g. anxiety, might provide more insights in this case study. One promising alternative to the M-DAS might be the Geneva Emotions Wheel (GEW) [40] that has convincing reliability and validity analyses [66].

Regarding our sample, in contrast to other AV studies only surveying valid drivers [16, 67], we included a broader age resulting in 18% of participants being below the age to obtain a driver’s license. One could argue that people who have never driven a car portray fundamentally different approaches towards driving than those who have. It remains an open question (for now) whether these are valuable insights for AV acceptance or not. Additionally, people below a certain age might not include relevant criteria in their assessment of AVs or might not be able to understand the question in the way adults do. This applies particularly to our outliers of 9 years old. However, a mean age below 35 years is a common sample characteristic in AV attitude research [23, 29, 68–70]. Additionally, our (non-randomised) sample consists of people interested in sciences and new technologies. High ratings on our acceptance and trust scales and low ratings on our fear scale thus reflect opinions of a very particularly population of people. Even if we had no concerns regarding the instruments, we would advise caution generalising from this sample. For further research designs, we advise comparisons between drivers and non-drivers, collecting data in regular operations opposed to single measurement points such as the Long Night of the Sciences, and samples outside the technically interested visitors of these special events.

Lastly, the operator might undermine expectations resulting from the term ‘automated vehicles’ that suggests a process completely detached from human interference. Strictly speaking, we did not investigate automated driving in its ideal form, but according to current technological advancements necessitating a human to oversee the machine. As apparent from the comments, these humans differ leaving us to consider the operator as a confounding variable in further analyses.

### Positioning findings and further research agenda

Our research project includes electric AVs enabling users to experience this technology immediately and physically. Our results are similar to those provided by studies using the same design [23, 29]. In the EU CityMobil2 project, Madigan et al. [23] applied the UTAUT variables to AVs using a sample with the same age mean and comparable gender distribution. Their almost identical intention to use scale yielded a higher Cronbach’s alpha (.90) than in our study (.83). In their EFA with varimax rotation, the scale produced a unique factor as in our EFA with oblique rotation. Madigan et al.’s [23] hedonic motivation measure can be compared to our amusement measure. It, too, produced a unique factor, but higher internal consistency (α = .87 compared to α = .77 in our study). The EU CityMobil2 projects offers a promising evaluation framework [71] well aligned with the approach presented in this article. Nordhoff et al. [29] reported similar ratings on the Van der Laan et al. [38] acceptance scale. However, in their principal component analysis the acceptance scale was split with items loading on the component ‘intention to use’ (items 1, 3, 5, and 8) and on unreported components (items 2, 4, 6, 7, and 9) [29]. This indicates uncertainty about the data structure created by this scale. Participants in Nordhoff et al. [29] critically assessed the slow speed of their AVs in a closed item mirrored by our analysis of our open item. Both Madigan et al. [23] and Nordhoff et al. [29] report generally positive attitudes towards AVs replicated in our study with slightly different methods. Other projects with physical experience of shared AVs have not provided reliable data yet [72, 73].

Comparisons with hypothetical surveys are difficult, because many studies provide scenarios different from ours, e.g. automation in privately owned cars bearing little resemblance with the shared, electric AVs in our study [16, 74]. Some studies do not specify an application scenario, but speak of AVs generally [13, 14, 24]. Those hypothetical surveys focusing on shared AVs use different measures, e.g. contextual acceptability and impaired driving [67], driving enjoyment and environmental concerns [75], or economic measures such as willingness to pay [11, 25, 26, 69]. Lastly, some studies model adoption rates based on model specifications of particular regions [76, 77]. Thus, our article—together with the two applications by Madigan et al. [23] and Nordhoff et al. [29]–addresses different questions than previous hypothetical studies limiting their comparability.

Lastly, we stress the open item’s valuable contribution to generating knowledge. The answers have not only improved our questionnaire for the main survey, but also enlightened us about qualitative aspects regarding experience on the ride with an AV. With the help of the comments, we identified the operator as a relevant confounder in acceptance of an AV. We also learnt more about driving style, technical features, and (unsettling) audio-visual feedback too specific to address with pre-built, scaled questionnaire items. Additionally, insights from the open item caution us to interpret the positive answers regarding acceptance, trust, perceived safety, and intention to use overcredulously. Thus, we agree with colleagues who have demonstrated the benefits of analysing open items in synergy with quantitative analyses [78], and promote the use of (more extensive) qualitative data collection in further research projects beyond simple classification and quantitative analysis [79, 80]. Such an approach might reformulate (narrow) definitions of constructs. Usefulness of and satisfaction with a system as subcategories of acceptance, for example, make sense within a framework that puts individual immediate experience with a system to the fore. This is even more the case when acceptance equates usage. From a sociological perspective, acceptance also comprises the social conditions surrounding the system in question—seeing technology not as a neutral force of change, but as a socio-technical arrangement of mutual influence. In our use case, AVs are the mobility version of digitalisation and automation substituting human labour and agency with machines and algorithms. These broader perspectives are arguably as important to acceptance as immediate contact with a system—if acceptance is defined and measured accordingly. Recognising with the present study that narrow definitions of attitudes only produce parts of what is needed for an informed debate about emerging technologies, we urge researchers to go beyond these technical views and embrace an interdisciplinary, mixed-methods approach towards AV research comparable to those in other fields [81, 82].

## Supporting information

S1 Appendix German questionnaire used in data conduction. (PDF)

S2 Appendix English questionnaire translated from German original. (PDF)

S1 Model Visual representation of the confirmatory factor analysis with nine latent variables. (PDF)

S1 Text Pattern matrices from Explorative factor analysis with oblique rotation for 31 items on nine scales with 7 factors and with 10 factors and corresponding Screeplot. (PDF)

S2 Text Screeplots from factor analyses with 4-item perceived safety scale (plus self-constructed general perceived safety item) and oblique rotation and corresponding pattern matrix. (PDF)

S3 Text Pattern matrices with oblique rotation for 12 emotions items and three and five retrieved factors and corresponding Screeplot. (PDF)

S1 Fig Screeplot for 9-item acceptance scale from EFA with oblique rotation. (PDF)

S2 Fig Screeplot for 3-item intention to use scale from EFA with oblique rotation. (PDF)

S3 Fig Screeplot for 3-item trust scale from EFA with oblique rotation. (PDF)
