# Supplementary material for: Assessing acceptance of electric automated vehicles after exposure in a realistic traffic environment
Source: PLoS One. 2019 May 2;14(5):e0215969. doi: 10.1371/journal.pone.0215969 (PMC6497263; doi:10.1371/journal.pone.0215969)
Supplement: S1 Appendix — (PDF) [file pone.0215969.s001.pdf]

CHARITÉ
UNIVERSITÄTSMEDIZIN BERLIN

S1 Appendix. German Questionnaire used in Data Conduction.

Fragebogen zu fahrerlosen Bussen an der Charité

Vielen Dank für Ihre Teilnahme an dieser Umfrage!
Bitte kreuzen Sie die jeweils zutreffende Angabe an bzw. schreiben die Information auf. Ihre Daten werden anonym verarbeitet. Bei Fragen wenden Sie sich bitte an die Ansprechperson.

**Wie alt sind Sie?**	Alter: ____________ Jahre		
**Bitte nennen Sie Ihr Geschlecht.**	☐ weiblich	☐ männlich	☐ keine Angabe
**Haben Sie einen PKW-Führerschein?**	☐ Ja	☐ Nein	

**Wie haben Sie sich während der Fahrt mit dem elektrischen, automatisierten Kleinbus gefühlt? Bitte geben Sie Ihre Einschätzung zu den folgenden Begriffen.**					
	Sehr schwach	Schwach	Teils/teils	Stark	Sehr stark
Gelangweilt	☐	☐	☐	☐	☐
Vergnügt	☐	☐	☐	☐	☐
Angeödet	☐	☐	☐	☐	☐
Überrascht	☐	☐	☐	☐	☐
Unbeteiligt	☐	☐	☐	☐	☐
Erschreckt	☐	☐	☐	☐	☐
Amüsiert	☐	☐	☐	☐	☐
Furchtsam	☐	☐	☐	☐	☐
Erstaunt	☐	☐	☐	☐	☐
Verblüfft	☐	☐	☐	☐	☐
Erheitert	☐	☐	☐	☐	☐
Ängstlich	☐	☐	☐	☐	☐

**Wie sicher haben Sie sich bei der Fahrt mit dem elektronischen, automatisierten Kleinbus gefühlt?**				
Sehr unsicher	unsicher	neutral	sicher	Sehr sicher
☐	☐	☐	☐	☐

Im Folgenden interessiert uns Ihre Meinung zu automatisierten Fahrzeuge im öffentlichen Personennahverkehr.

	Stimme nicht zu	Stimme eher nicht zu	Teils-teils	Stimme eher zu	Stimme zu
Wenn ich Zugang zu einem automatisierten Fahrzeug hätte, dann würde ich es vermutlich nutzen.	☐	☐	☐	☐	☐
Ich würde mich sicher fühlen, wenn ich automatisierte Fahrzeuge nutze.	☐	☐	☐	☐	☐
Ich vertraue automatisierten Fahrzeugen, weil sie meine Interessen berücksichtigen.	☐	☐	☐	☐	☐
Sobald automatisierte Fahrzeuge verfügbar sind, plane ich, eines in den nächsten Monaten zu nutzen.	☐	☐	☐	☐	☐
Automatisierte Fahrzeuge zu nutzen, verringert das Unfallrisiko.	☐	☐	☐	☐	☐
Automatisierte Fahrzeuge halten, was sie versprechen und zusagen.	☐	☐	☐	☐	☐
Automatisierte Fahrzeuge sind vertrauenswürdig.	☐	☐	☐	☐	☐
Ich glaube, dass das Nutzen von automatisierten Fahrzeugen gefährlich ist.	☐	☐	☐	☐	☐
Wenn ich Zugang zu einem automatisierten Fahrzeug hätte, dann würde ich es auf jeden Fall nutzen.	☐	☐	☐	☐	☐
Automatisierte Fahrzeuge zu nutzen, erfordert erhöhte Aufmerksamkeit.	☐	☐	☐	☐	☐

**Meine Beurteilung für automatisierte Fahrzeuge lautet:**		
nützlich	☐ ☐ ☐ ☐ ☐	nutzlos
angenehm	☐ ☐ ☐ ☐ ☐	unangenehm
schlecht	☐ ☐ ☐ ☐ ☐	gut
erleichternd	☐ ☐ ☐ ☐ ☐	lästig
effektiv	☐ ☐ ☐ ☐ ☐	überflüssig
ärgerlich	☐ ☐ ☐ ☐ ☐	erfreulich
hilfreich	☐ ☐ ☐ ☐ ☐	wertlos
nicht wünschenswert	☐ ☐ ☐ ☐ ☐	wünschenswert
anregend	☐ ☐ ☐ ☐ ☐	einschläfernd

Haben wir etwas nicht beachtet? Bitte geben Sie uns Ihre Anmerkungen zum Projekt oder den Fahrzeugen.
