# Supplementary material for: Assessing acceptance of electric automated vehicles after exposure in a realistic traffic environment
Source: PLoS One. 2019 May 2;14(5):e0215969. doi: 10.1371/journal.pone.0215969 (PMC6497263; doi:10.1371/journal.pone.0215969)
Supplement: S2 Appendix — (PDF) [file pone.0215969.s002.pdf]

S2 Appendix. English Questionnaire Translated from German Original.

Questionnaire about driverless busses at the Charité

Thank you very much for participating in this survey!
Please tick the box next to the statement you find most appropriate or make the respective note. Your data will be processed anonymously. Please consult the contact person if you have any questions.

**How old are you?**	Age: ____________ Years		
**Please indicate your gender.**	☐ female	☐ male	☐ prefer not to say
**Do you have a driver's license for passenger cars?**	☐ Yes	☐ No	

**How did you feel on the ride with the electric automated bus? Please give your evaluation for the following terms.**					
	Very weak	Weak	Neutral	Strong	Very strong
Bored	☐	☐	☐	☐	☐
Silly	☐	☐	☐	☐	☐
Bored stiff	☐	☐	☐	☐	☐
Surprised	☐	☐	☐	☐	☐
Uninvolved	☐	☐	☐	☐	☐
Scared	☐	☐	☐	☐	☐
Amused	☐	☐	☐	☐	☐
Fearful	☐	☐	☐	☐	☐
Amazed	☐	☐	☐	☐	☐
Astonished	☐	☐	☐	☐	☐
Fun-loving	☐	☐	☐	☐	☐
Afraid	☐	☐	☐	☐	☐

**How safe did you feel on the ride with the electric automated bus?**				
Very unsafe	Unsafe	Neutral	Safe	Very safe
☐	☐	☐	☐	☐

CHARITÉ
UNIVERSITÄTSMEDIZIN BERLIN

In the following, we are interested in your opinion about automated vehicles in local public transport.

	Disagree	Some-what disagree	Un-decided	Some-what agree	Agree
Assuming I had access to an autonomous vehicle, I intend to use it.	☐	☐	☐	☐	☐
I feel safe when using autonomous vehicles.	☐	☐	☐	☐	☐
I trust autonomous vehicles, because they keep my best interests in mind.	☐	☐	☐	☐	☐
If autonomous vehicles are available, I plan to use one in the next months.	☐	☐	☐	☐	☐
Using autonomous vehicles decreases the accident risk.	☐	☐	☐	☐	☐
Autonomous vehicles keep promises and commitments.	☐	☐	☐	☐	☐
Autonomous vehicles are trustworthy.	☐	☐	☐	☐	☐
I believe using autonomous vehicles is dangerous.	☐	☐	☐	☐	☐
Given I had access to an autonomous vehicle, I predict that I would use it.	☐	☐	☐	☐	☐
Using autonomous vehicles requires increased attention.	☐	☐	☐	☐	☐

**My judgements of automated vehicles are:**		
useful	☐ ☐ ☐ ☐ ☐	useless
pleasant	☐ ☐ ☐ ☐ ☐	unpleasant
bad	☐ ☐ ☐ ☐ ☐	good
nice	☐ ☐ ☐ ☐ ☐	annoying
effective	☐ ☐ ☐ ☐ ☐	superfluous
irritating	☐ ☐ ☐ ☐ ☐	likeable
assisting	☐ ☐ ☐ ☐ ☐	worthless
undesirable	☐ ☐ ☐ ☐ ☐	desirable
raising alertness	☐ ☐ ☐ ☐ ☐	sleep-inducing

Did we leave something out? Please give us your comments about the project or the vehicles.
