# Supplementary material for: Assessing acceptance of electric automated vehicles after exposure in a realistic traffic environment
Source: PLoS One. 2019 May 2;14(5):e0215969. doi: 10.1371/journal.pone.0215969 (PMC6497263; doi:10.1371/journal.pone.0215969)

S1 Model. Visual representation of the confirmatory factor analysis with nine latent variables.

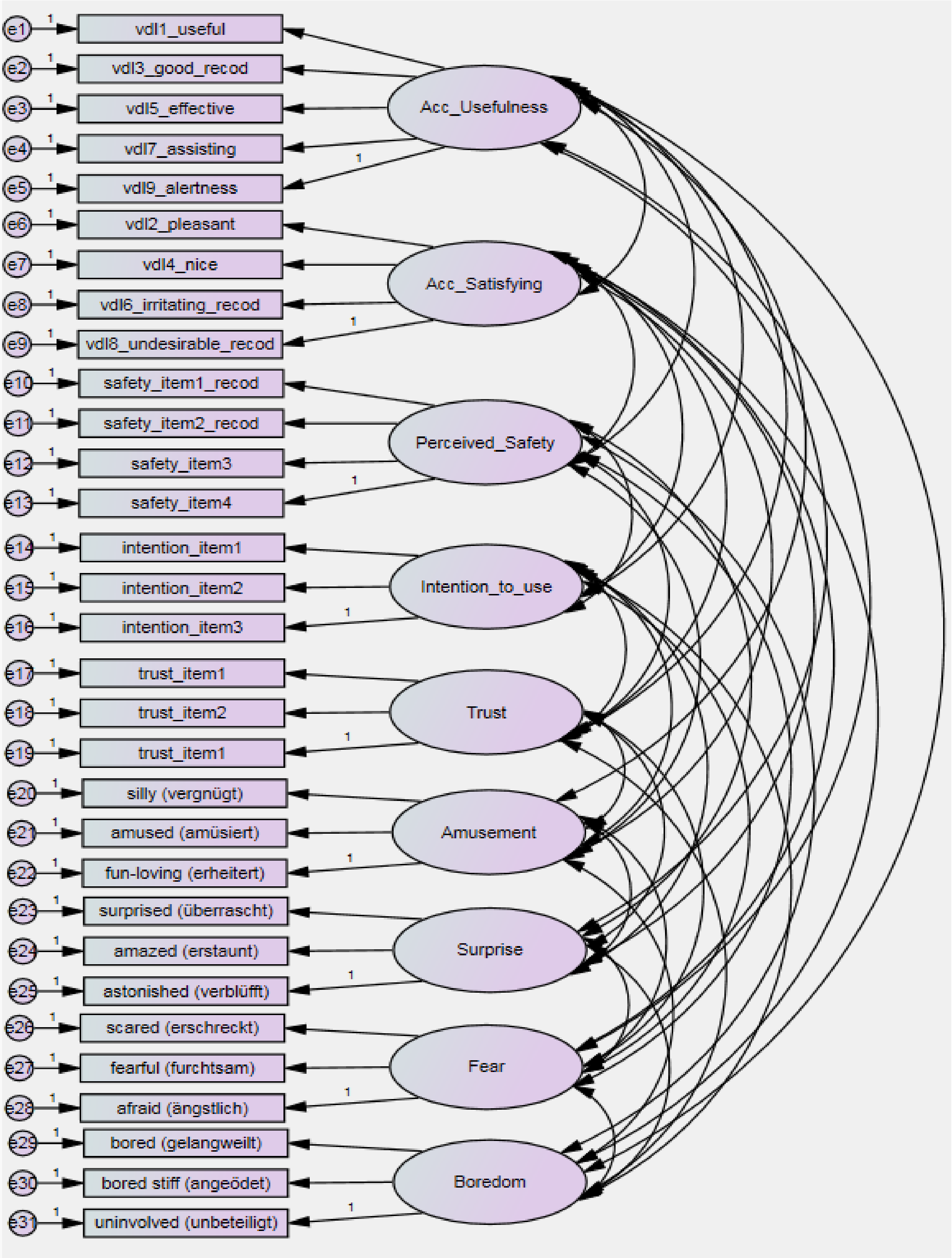

Supplement: S1 Model — (PDF) [file pone.0215969.s003.pdf]
