# Supplementary material for: Assessing acceptance of electric automated vehicles after exposure in a realistic traffic environment
Source: PLoS One. 2019 May 2;14(5):e0215969. doi: 10.1371/journal.pone.0215969 (PMC6497263; doi:10.1371/journal.pone.0215969)

S1 Text Table A. Pattern Matrix with Oblique Rotation for 31 Items on Nine Scales with 7 Factors.

**Item**	**Factors**						
	**1**	**2**	**3**	**4**	**5**	**6**	**7**
Useful – useless [a]	.87						
Effective – superfluous [a]	.84						
Assisting – worthless [a]	.83						
Pleasant – unpleasant [b]	.68						
Nice – annoying [b]	.67						
Bad – good [†, a]	.64						
Undesirable – desirable [†, b]	.64						
Irritating – likeable [†, b]	*.47*						
Raising alertness – sleep-inducing [a]	*.47*						
Fun-loving [f]		.77					
Amused [f]		.76					
Silly [f]		.70					
Uninvolved [i]		-.54					
Fearful [g]			.87				
Afraid [g]			.81				
Scared [g]			.78				
AVs keep promises and commitments. [e]				.84			
Using AVs decreases the accident risk. [c]				.59			
AVs are trustworthy. [e]				.53			
I believe using AVs is dangerous. [†, c]				.52			
I trust AVs, because they keep my best interests in mind. [e]				*.43*			

**S1 Text Table A continued.**							
**Item**	**Factors**						
	**1**	**2**	**3**	**4**	**5**	**6**	**7**
Given I had access to AV, I predict that I would use it. [d]					-.78		
If AVs are available, I plan to use one in the next months. [d]					-.73		
Assuming I had access to an AV, I intend to use it. [d]					-.69		
I feel safe when using AVs. [c]					-.59		
Using AVs requires increased attention. [†, c]					*.48*		
Amazed [h]						.90	
Astonished [h]						.87	
Surprised [h]						.81	
Bored stiff [i]							.74
Bored [i]							.61

Only factor loadings above an absolute value of 0.50 or the highest loading of the item (*italics*) are displayed.

[†] items are reversed.

[a] acceptance usefulness, [b] acceptance satisfying, [c] perceived safety, [d] intention to use, [e] trust, [f] amusement, [g] fear, [h] surprise, [i] boredom

S1 Text Table B. Pattern Matrix with Oblique Rotation for 31 Items on Nine Scales with 10 Factors.

Item	Factors									
	1	2	3	4	5	6	7	8	9	10
Effective – superfluous [a]	.88									
Useful – useless [a]	.88									
Assisting – worthless [a]	.82									
Pleasant – unpleasant [b]	.58									
Nice – annoying [b]	.53								*-.43*	
Undesirable – desirable [†, b]	*.46*									
Amused [f]		.84								
Silly [f]		.81								
Fun-loving [f]		.64								
Fearful [g]			.88							
Afraid [g]			.86							
Scared [g]			.72						*-.39*	
AVs keep promises and commitments. [e]				.91						
I trust AVs, because they keep my best interests in mind. [e]				*.47*	*-.34*				*-.43*	
AVs are trustworthy. [e]				*.45*						
Given I had access to AV, I predict that I would use it. [d]					-.80					
If AVs are available, I plan to use one in the next months. [d]					-.74					
Assuming I had access to an AV, I intend to use it. [d]					-.57					
I believe using AVs is dangerous. [†, c]				*.39*	.54		*.34*			
I feel safe when using AVs. [c]				*.33*	*-.46*		*.34*			

S1 Text Table B continued.										
**Item**	**Factors**									
	**1**	**2**	**3**	**4**	**5**	**6**	**7**	**8**	**9**	**10**
Amazed [h]						.89				
Astonished [h]						.87				
Surprised [h]						.86				
Bored stiff [i]							.81			
Bored [i]							.63			
Raising alertness – sleep-inducing [a]							*-.40*		*-.39*	
Uninvolved [i]								.94		
Irritating – likeable [†, b]									*-.27*	
Using AVs decreases the accident risk. [c]										.82
Using AVs requires increased attention. [†, c]										.76
Bad – good [†, a]	*.32*								*-.35*	*.44*

Only factor loadings above an absolute value of 0.50 or the highest loading of the item (*italics*) are displayed. Crossloadings above an absolute value of 0.32 are also displayed (*italics*)

[†] items are reversed.

[a] acceptance usefulness, [b] acceptance satisfying, [c] perceived safety, [d] intention to use, [e] trust, [f] amusement, [g] fear, [h] surprise, [i] boredom

S1 Text Fig. Screeplot from Factor Analysis with 31 items and Oblique Rotation.

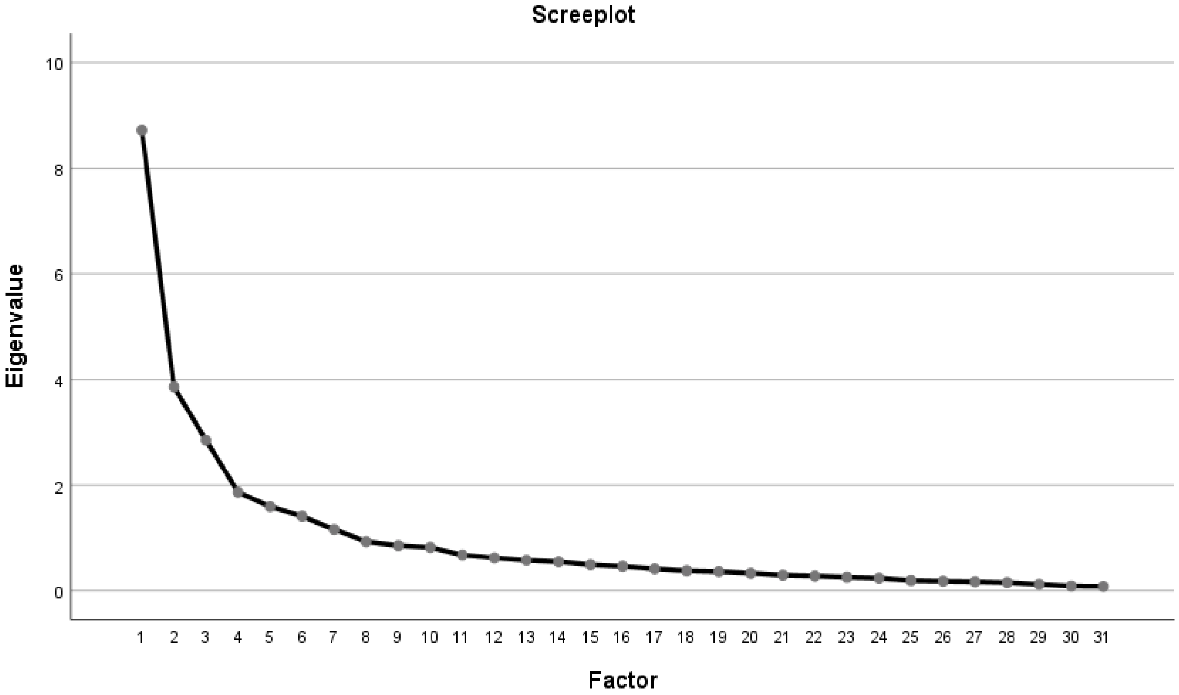

Supplement: S1 Text — (PDF) [file pone.0215969.s004.pdf]
