# Supplementary material for: Assessing acceptance of electric automated vehicles after exposure in a realistic traffic environment
Source: PLoS One. 2019 May 2;14(5):e0215969. doi: 10.1371/journal.pone.0215969 (PMC6497263; doi:10.1371/journal.pone.0215969)
Supplement: S2 Text — (PDF) [file pone.0215969.s005.pdf]

S2 Text Fig A. Screeplot from Factor Analysis with 4-Item Perceived Safety Scale and Oblique Rotation.

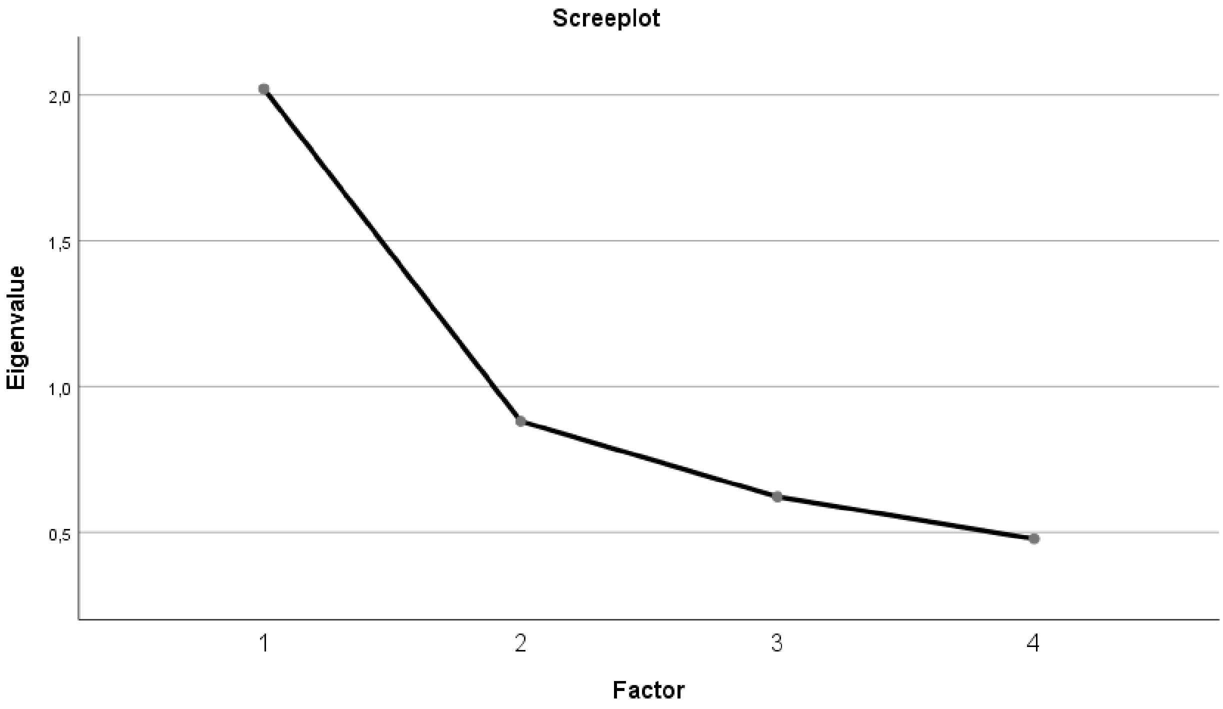

S2 Text Fig B. Screeplot from Factor Analysis with 4-Item Perceived Safety Scale plus Self-Constructed General Perceived Safety Item and Oblique Rotation.

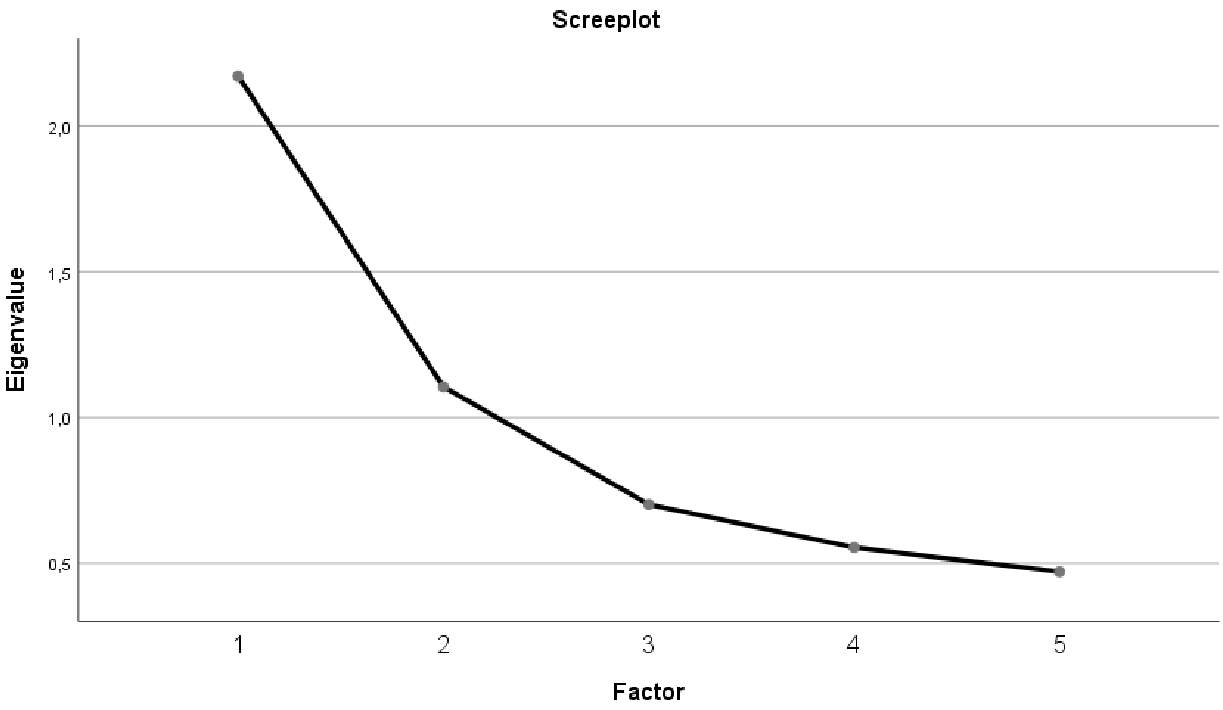

S2 Text Table. Pattern Matrix with Oblique Rotation for Perceived Safety Scale plus Self-Constructed Item.

**Item**	**Component 1**	**Component 2**
Using AVs requires increased attention. (item 2)	**.82**	.34
I believe using AVs is dangerous. (item 1)	**.73**	-.26
Using AVs decreases the accident risk. (item 4)	**.65**	-.28
How safe did you feel on the ride? (self-constructed)	-.12	**-.86**
I feel safe when using AVs. (item 3)	.30	**-.70**

Item loadings with an absolute value above .50 are displayed in bold. Items 1 and 2 are reversed
