# Supplementary material for: Assessing acceptance of electric automated vehicles after exposure in a realistic traffic environment
Source: PLoS One. 2019 May 2;14(5):e0215969. doi: 10.1371/journal.pone.0215969 (PMC6497263; doi:10.1371/journal.pone.0215969)

S3 Text Table A. Pattern Matrix with Oblique Rotation for 12 Emotions Items and Three Retrieved Factors.

**Item**	**Factor 1**	**Factor 2**	**Factor 3**
Amused	**.78**	.08	-.00
Silly	**.77**	-.00	-.08
Fun-loving	**.74**	.16	-.00
Bored	**-.73**	.12	.18
Bored stiff	**-.64**	.24	.08
Uninvolved	**-.51**	.02	-.15
Fearful	.01	**.92**	.04
Scared	-.01	**.81**	-.05
Afraid	.00	**.79**	-.06
Amazed	.04	-.03	**-.94**
Astonished	.12	.08	**-.87**
Surprised	-.09	.03	**-.81**

Item loadings with an absolute value above .50 are displayed in **bold**; crossloadings above an absolute value of 0.32 are displayed in *italics*.

S3 Text Table B. Pattern Matrix with Oblique Rotation for 12 Emotions Items and Five Retrieved Factors.

**Item**	**Factor 1**	**Factor 2**	**Factor 3**	**Factor 4**	**Factor 5**
Amused	**.86**	-.01	.04	-.08	.10
Fun-loving	**.82**	.06	-.01	.05	-.09
Silly	**.73**	-.07	-.06	-.13	-.07
Fearful	.05	**.90**	.03	.11	-.05
Afraid	-.19	**.87**	.03	-.27	.00
Scared	.16	**.76**	.06	.22	.05
Amazed	-.05	.00	**-.91**	-.20	.10
Surprised	-.01	-.02	**-.87**	-.27	-.20
Astonished	.11	.09	**-.84**	-.14	.15
Bored stiff	-.08	.08	.01	**.84**	.13
Bored	-*.32*	.02	.11	**.70**	.04
Uninvolved	-.03	-.02	-.02	.11	**.96**

Item loadings with an absolute value above .50 are displayed in **bold**; crossloadings above an absolute value of 0.32 are displayed in *italics*.

S3 Text Fig. Screeplot for 12 Emotions Items from EFA with Oblique Rotation.

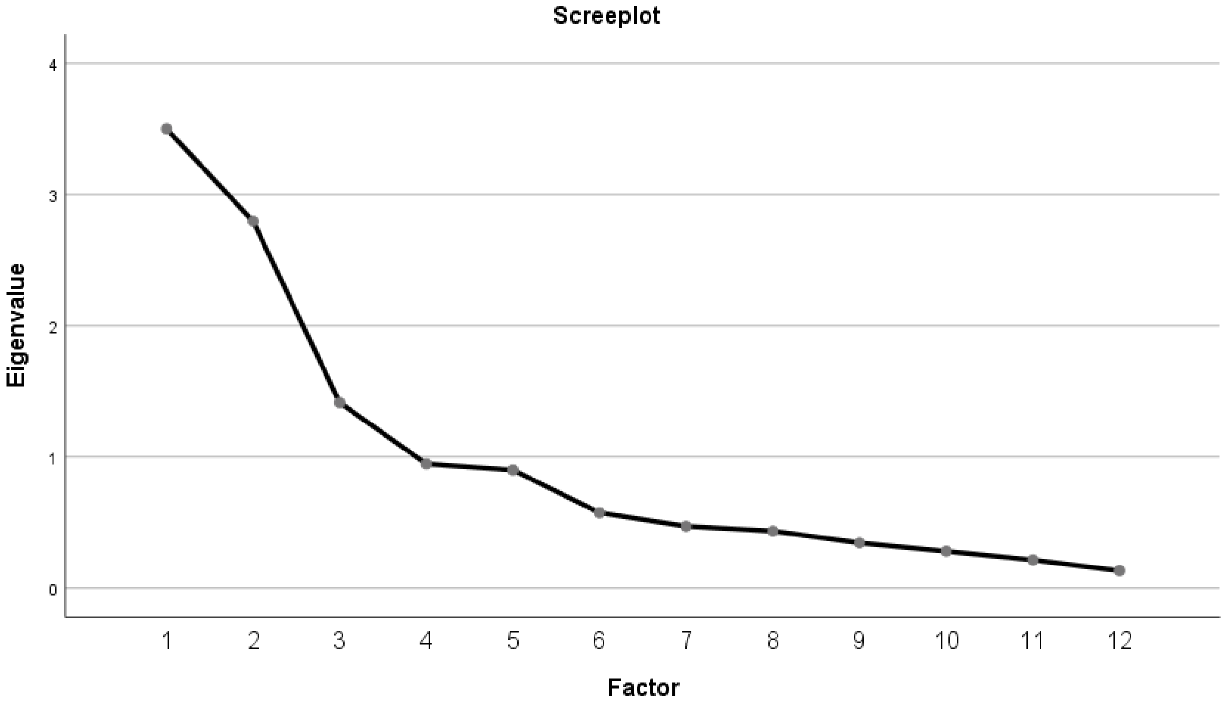

Supplement: S3 Text — (PDF) [file pone.0215969.s006.pdf]
