# Supplementary figures and images for: Assessing acceptance of electric automated vehicles after exposure in a realistic traffic environment
Source: PLoS One. 2019 May 2;14(5):e0215969. doi: 10.1371/journal.pone.0215969 (PMC6497263; doi:10.1371/journal.pone.0215969)

**S1 Fig. Screeplot for 9-item Acceptance Scale from EFA with Oblique Rotation.**

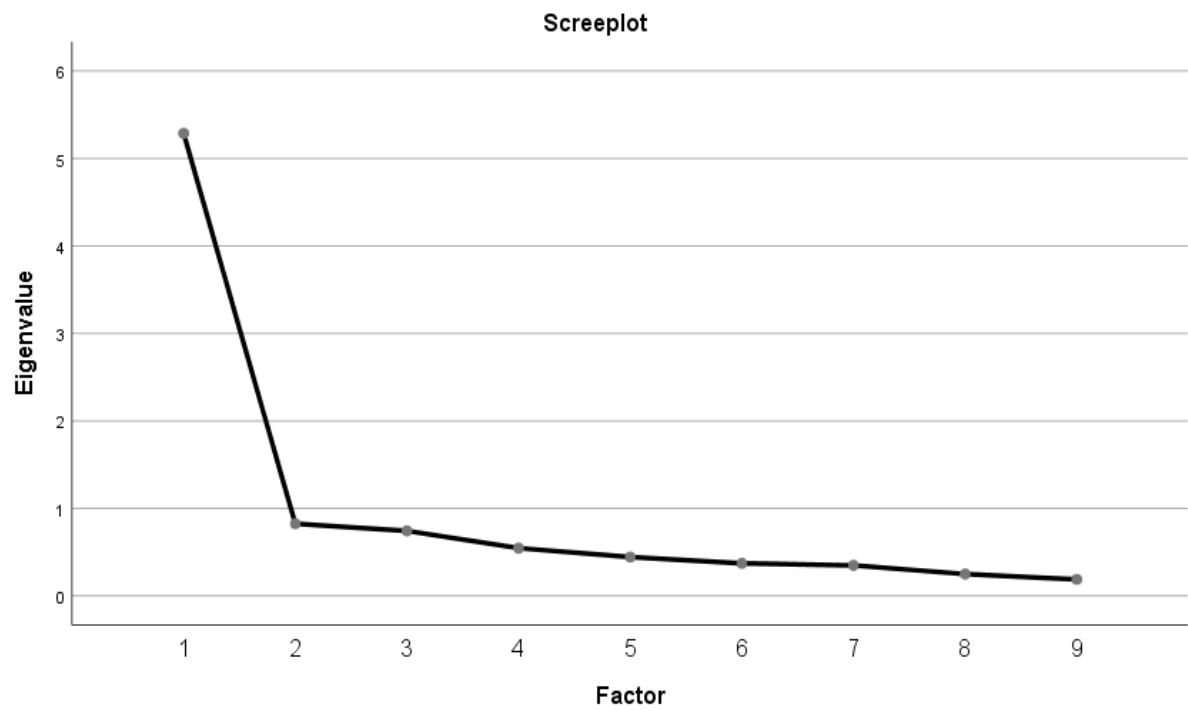

Supplement: S1 Fig — (PDF) [file pone.0215969.s007.pdf]

**S2 Fig. Screeplot for 3-item Intention to Use Scale from EFA with Oblique Rotation.**

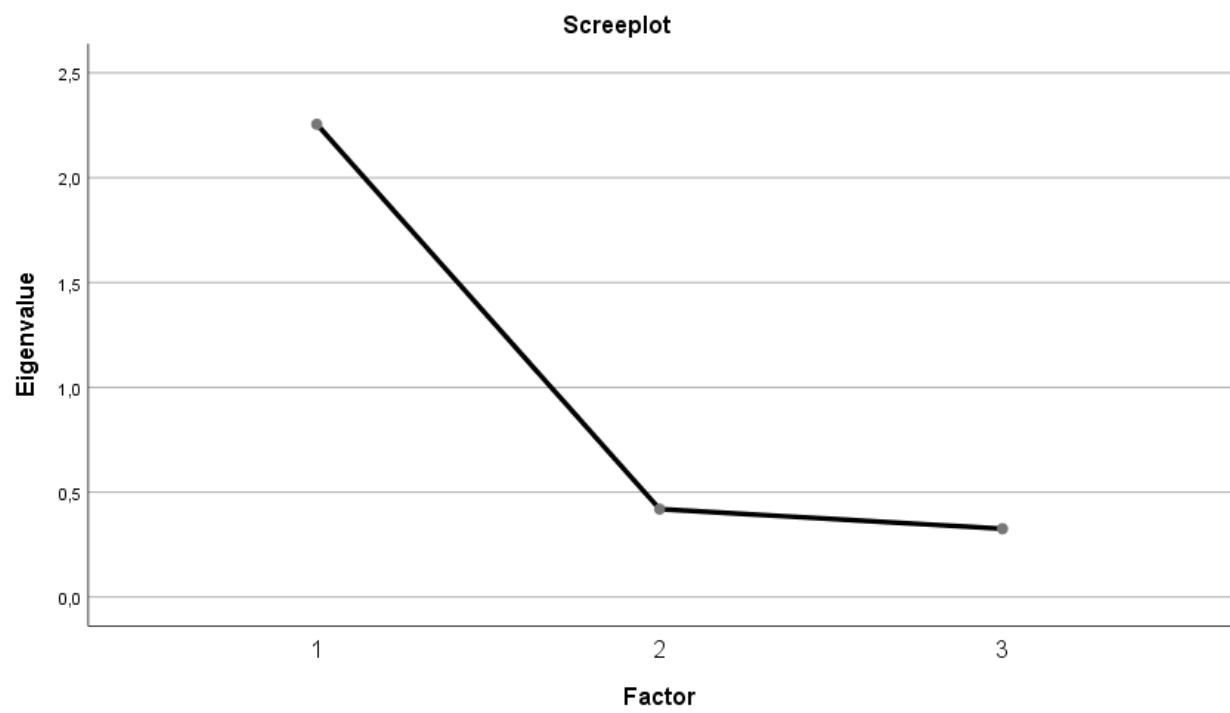

Supplement: S2 Fig — (PDF) [file pone.0215969.s008.pdf]

S3 Fig. Screeplot for 3-item Trust Scale from EFA with Oblique Rotation.

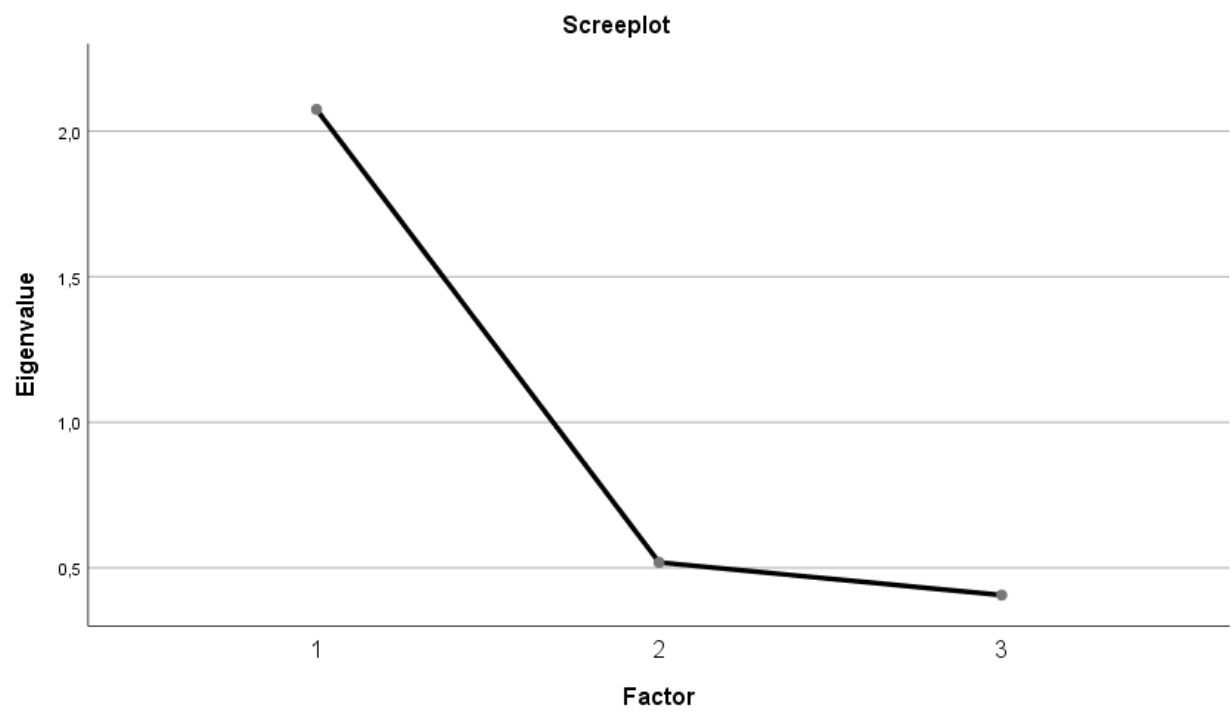

Supplement: S3 Fig — (PDF) [file pone.0215969.s009.pdf]
